# Broadband Near-Infrared Emission from Bi/Cr Co-Doped Aluminosilicate Glasses

**DOI:** 10.3390/mi15091093

**Published:** 2024-08-29

**Authors:** Shiwen Song, Min Zhang

**Affiliations:** School of Physics and Astronomy, China West Normal University, Nanchong 637002, China; songshiwen97@163.com

**Keywords:** bismuth, aluminosilicate glasses, chromium, broadband NIR emission

## Abstract

Bismuth-doped aluminosilicate glass has garnered significant attention due to its unique ultra-wide luminescence properties in the near-infrared (NIR) band. Enhancing the NIR luminescence of Bi-doped glass remains challenging. To achieve Bi-doped glass with more excellent luminescent properties, a series of Bi/Cr co-doped glasses were prepared, and the optical and structural properties of the samples were observed. The results indicate that low-concentration Cr doping broadens the luminescence range of Bi/Cr co-doped glass samples. The luminescence peak of Bi in the samples is at 1230 nm, while the peak of Cr is around 804 nm. The addition of an appropriate amount of Bi_2_O_3_ can enhance the NIR luminescence of Bi and Cr in the sample, realizing the energy conversion between Bi and Cr. Bi/Cr co-doped is a novel approach for achieving broadband NIR luminescence in glass materials.

## 1. Introduction

With the rapid arrival of the fifth-generation communication technology (5G), the development of communications has placed higher demands on the information transmission capabilities of optical communication systems. The near-infrared (NIR) band, spanning from 1000 to 1600 nm, represents a crucial window for optical communications [1,2,3]. However, traditional optical amplifiers struggle to cover this entire spectrum. Traditional optical fiber amplifiers typically use rare-earth-doped materials, which generally exhibit narrowband emission [4,5]. For instance, the widely utilized erbium-doped fiber amplifier in the traditional sense can only cover the 1530 to 1600 nm range, owing to its special 4f-4f forbidden transition [6]. Consequently, new optical fiber materials that can cover the 1000 to 1600 nm range are urgently needed.

In recent years, bismuth-doped glasses have been widely applied as gain materials in tunable fiber lasers operating in the NIR spectral region due to their long fluorescence lifetime and ultra-broadband NIR emission characteristics [7,8,9]. Additionally, Bi-doped glass fibers have achieved laser output in the range of 1000–1600 nm [10,11,12]. Consequently, Bi-doped glasses are considered promising candidates for next-generation broadband fiber amplifiers and lasers to address the capacity demands of modern communication systems. Despite significant advancements, few Bi-doped glass materials achieve both high luminescence intensity and broad spectral range in the NIR region. Recently, various methods have been tried in the search for efficient luminescence of Bi-doped materials. The topochemical reduction of bismuth using reducing agents such as AlN, Si_3_N_4_, and SiC in Bi-doped germanate glass has successfully expanded Bi luminescence across the entire NIR region [13,14,15]. However, achieving a delicate balance between the reducing and glass melting processes is challenging. The reduction of Bi^3+^ ions to low-valent Bi luminescent centers can also be achieved by high-energy irradiation through light-activated redox, although this often results in the creation of defects that can complicate experimental procedures [13]. Therefore, it is necessary to find new methods to enhance the NIR luminescence of Bi ions and broaden the luminescence range.

The transition metal ion Cr^3+^ is a highly effective NIR emission activator in luminescent materials [16,17,18]. The 3d^3^ transitions of Cr^3+^ ions are highly sensitive to changes in the crystal field, enabling wavelength adjustment [19]. Moreover, in a weak crystal field environment, Cr^3+^ ions undergo the ^4^T_2_ → ^4^A_2_ transition, covering the NIR region of 650~1400 nm [20]. To date, Bi NIR luminescence properties have been extensively studied in phosphate [21,22], silicate [23,24], borate [12] and germanate [25,26] glass systems. Silicate glass exhibits more stable chemical and mechanical properties compared to other glass matrices. In addition, silicate glass provides a weak crystalline environment, which can make Cr^3+^ ions produce NIR emission. A large number of experiments have demonstrated that the NIR luminescence of Bi can be optimized by adjusting the Al content, and that CaO effectively lowers the glass melting point. Therefore, this study combines these advantages to prepare optimized samples.

In this study, Bi/Cr co-doped aluminosilicate glass was successfully prepared by the high-temperature melting method. At low Cr_2_O_3_ doping concentrations, Bi/Cr luminescence centers coexist, and the NIR luminescence intensity can be tuned by varying the Bi doping concentration. The NIR luminescence properties and energy transfer mechanism of Bi/Cr co-doped glass were systematically investigated.

## 2. Experimental Procedures

### 2.1. Materials Preparation

Glass samples were synthesized by conventional melt-quenching technique with compositions of (60−x)SiO_2_-10Al_2_O_3_-30CaO-0.5Bi_2_O_3−x_Cr_2_O_3_ (in mol%, x = 0, 0.02, 0.04, 0.06, 0.08), (60−y)SiO_2_-10Al_2_O_3_-30CaO-yBi_2_O_3_-0.1Cr_2_O_3_ (in mol%, y = 0, 0.5, 1) and (60−z)SiO_2_-10Al_2_O_3_-30CaO-zBi_2_O_3_-0.005Cr_2_O_3_ (in mol%, z = 0, 0.5, 1, 1.5, 2). Commercial SiO_2_, Al_2_O_3_, CaO, Bi_2_O_3_, and Cr_2_O_3_ were employed as starting materials of high chemical purity (99.99%). The powders were weighted accurately and ground thoroughly in an agate mortar. The well-ground stoichiometric chemicals were put into an alumina crucible and melted at 1600 °C in air for 1.5 h by a high temperature lifting furnace. The melt was poured onto a preheated (~200 °C) stainless-steel plate and pressed by another plate to improve the cooling rate and form solid glasses. All glasses were annealed at 750 °C for 5 h to remove thermal strains. Samples were polished for further optical characterization, with a size of 10 × 10 × 1 mm^3^.

### 2.2. Characterization

The absorption spectra for all samples were measured by an ultraviolet-visible-near-infrared spectrophotometer (UV–VIS–NIR, UV–3600i Plus, Shimadzu, Tokyo, Japan) in the range of 300–1200 nm. The fluorescence spectra were recorded by a steady-state fluorescence spectroscopy spectrometer (FLS1000, Edinburgh Instruments, Edinburgh, UK). The densities of glass were tested by the Archimedes principle with distilled water as the medium (MDJ–300T, Beijing, China). The Fourier transform infrared (FTIR) spectra were performed on a FTIR spectrometer (Nicolet 6700, Thermo Fisher Scientific, Waltham, MA, USA) by dispersing the sample powders uniformly on KBr particles within the range 400–2000 cm^−1^. X-ray diffraction spectroscopy (XRD) of the samples were performed by an X-ray diffractometer (TD–3500, Dandong Tongda, Dandong, China). 

## 3. Results and Discussion

**Figure 1:**
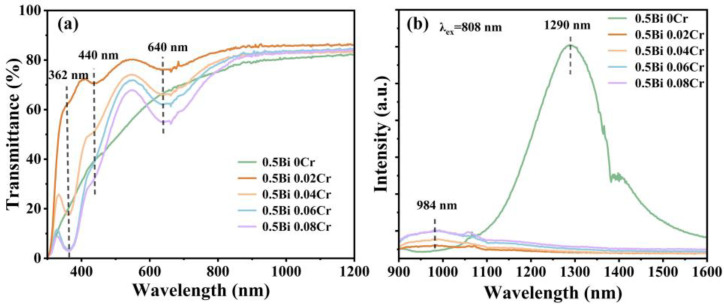
(**a**) Transmittance spectra of 0.5Bi_2_O_3−x_Cr_2_O_3_ (x = 0, 0.02, 0.04, 0.06, 0.08) glass samples. (**b**) The NIR emission spectra of 0.5Bi_2_O_3−x_Cr_2_O_3_ glasses under the excitation of 808 nm.

Figure 1a shows the transmittance spectra for 0.5Bi_2_O_3_−xCr_2_O_3_ (x = 0, 0.02, 0.04, 0.06, 0.08) glass samples. Three absorption peaks at ~362, ~440 and 640 nm are noticed in the visible range. According to previous studies, the absorption bands at 362 nm are assigned to the ^4^A_2_ → ^2^A_1_ transition of Cr^6+^, while the peaks at 440 and 640 nm are contributed by the ^4^A_2_ → ^4^T_1_ and ^4^A_2_ → ^4^T_2_ transitions of Cr^3+^ [27,28,29]. With the increasing concentration of Cr_2_O_3_, the absorbance at 362 nm, 440 nm, and 640 nm gradually increases. Notably, no significant Bi-related characteristic absorption peaks are detected in Figure 1a, suggesting a limited presence of Bi NIR emission centers in the glass samples. 

The NIR emission spectra for 0.5Bi_2_O_3_−xCr_2_O_3_ (x = 0, 0.02, 0.04, 0.06, 0.08) glass samples under 808 nm excitation at room temperature are portrayed in Figure 1b. For the Bi-doped glass sample, the emission peak located at 1290 nm might be assigned to the ^2^D_3/2_ → ^4^S_3/2_ transition of Bi^0^ [30,31]. However, with the addition of chromium, the Bi-related NIR luminescence peak vanishes, and a new peak appears at 984 nm, which increases in intensity with higher chromium content. According to previous reports, the luminescence of 984 nm is from the ^4^T_2_ → ^4^A_2_ transition of Cr^3+^ [32]. Under the 808 nm, part of the energy is absorbed by a number of Cr^3+^ ions and consumed through non-radiative transition processes, significantly reducing the energy available for Bi ions, leading to the quenching of the 1290 nm emission peak. Therefore, the emission peak at 1290 nm of Bi^0^ that was quenched may be due to the high Cr_2_O_3_ concentration.

**Figure 2:**
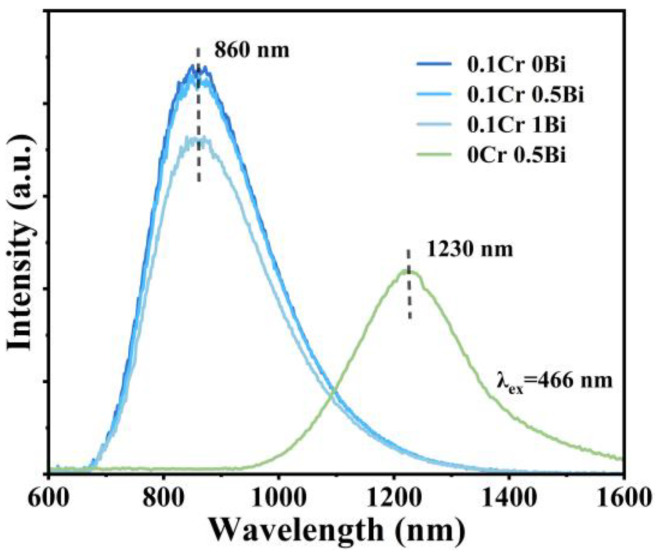
The NIR emission spectra of xBi_2_O_3_-0.1Cr_2_O_3_ (x = 0, 0.5, 1) and 0.5Bi_2_O_3_-0Cr_2_O_3_ glasses under the excitation of 466 nm.

To further confirm the above result, the concentration of Cr_2_O_3_ was increased to 0.1 mol, and a series of xBi_2_O_3_-0.1Cr_2_O_3_ (x = 0, 0.5, 1) samples were prepared. The NIR luminescence spectra of glass samples under 466 nm excitation are shown in Figure 2. The results confirm that Bi luminescence is quenched at a Cr_2_O_3_ concentration of 0.1 mol, consistent with the observations in Figure 1b. This behavior aligns with previous studies, suggesting a strong dependence on Cr_2_O_3_ doping concentration. 

Considering that too high a concentration of Cr ions can quench the luminescence of Bi, a low concentration of Cr was selected for doping. In structurally stable glass samples, the Bi ion concentration is also one of the important factors affecting the luminescence performance of the glass samples. In order to achieve co-doping of Bi and Cr for enhanced joint luminescence and to further explore the optimal glass component, the doping concentration of Cr_2_O_3_ was maintained at 0.005 mol, while varying the doping concentrations of Bi_2_O_3_ were used to study the structural properties and luminescence behavior of the glasses.

**Figure 3:**
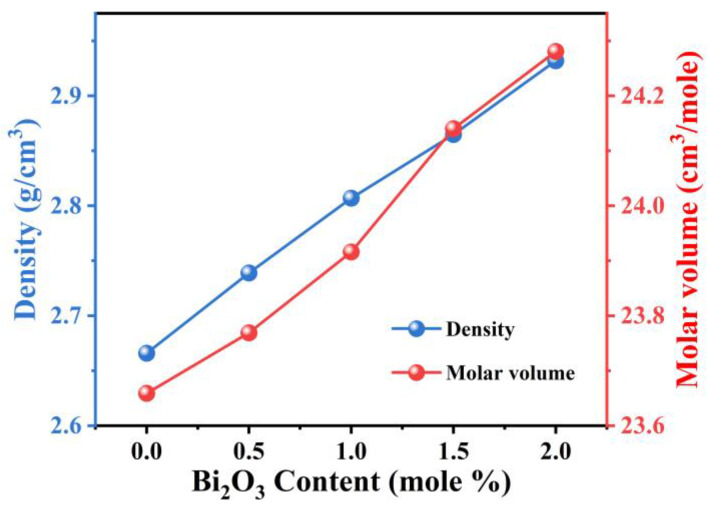
Variation of density and molar volume with the content of Bi_2_O_3_.

The variation of density and molar volume with the content of is shown in Figure 3. The measured density of samples increased monotonically from 2.67 g/cm^3^ to 2.93 g/cm^3^ as Bi_2_O_3_ replaced SiO_2_. The increasing density of samples may be due to the higher molecular weight of Bi_2_O_3_ than of SiO_2_ [33]. Molar volume ($V_{m}$), as an important parameter to describe the compactness of glass, can be obtained by the following formula:

(1) $$V_{m}=\sum x_{i}\cdot M_{i}/\rho$$

Here, $x_{i}$ is the molar fraction of oxide composition, $M_{i}$ is the molecular mass of oxide of nominal compositions of sample, ρ is the density of glass.

The molar volume of the samples can be calculated by Equation (1) to show a monotonically decreasing trend, which is exactly the same as the density trend in the samples. Normally, density and molar volume show opposite trends, but the result of the present work is anomalous. The increase in molar volume with increasing Bi_2_O_3_ content is most likely attributed to the replacement of a smaller ionic radius Si by a larger ionic radius Bi, which points to the change in the structure of glass [34,35]. 

**Figure 4:**
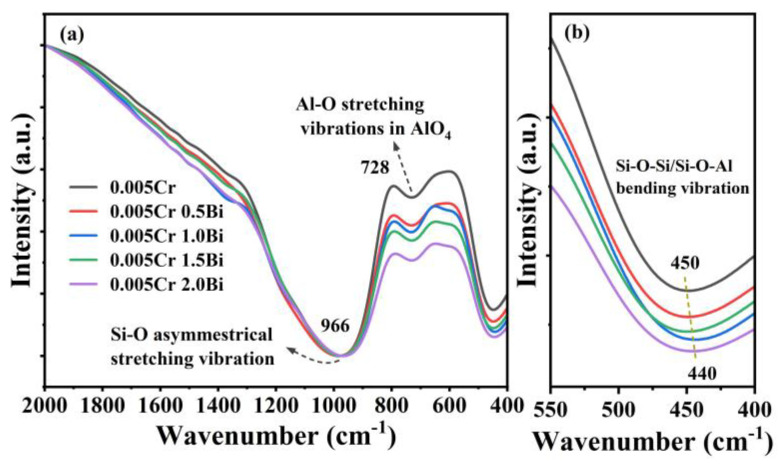
(**a**) FTIR spectra of xBi_2_O_3_-0.005Cr_2_O_3_ glasses (x = 0, 0.5, 1, 1.5, 2). (**b**) Amplified FTIR in the range of 400–550 cm^−1^ showing the variation of the Si-O-Al vibration peaks with the concentration of Bi_2_O_3_.

FTIR spectroscopy can be applied to observe chemical bonds and unit structure information in glass. Figure 4a is the FTIR spectra of the xBi_2_O_3_-0.005Cr_2_O_3_ glasses. Three obvious absorption peaks can be noticed in the range of 400–2000 cm^−1^ in Figure 4a. The Si-O asymmetrical stretching vibration peak at 966 cm^−1^ is dominant, and the stability of the relative intensity of the absorption peak indicates that the number of non-bridging oxygens (NBOs) remains basically unchanged, which also implies that the silicon network has not depolymerized. The stretching vibrations of Al-O (~728 cm^−1^) in the isolated AlO_4_ structural unit increase as the bismuth concentration increases. The absorption peak at 450 cm^−1^ gradually shifted from 450 cm^−1^ to 440 cm^−1^, which may be the formation of new Si-O-Al links in the silicon network structure, as shown in Figure 4b. 

**Figure 5:**
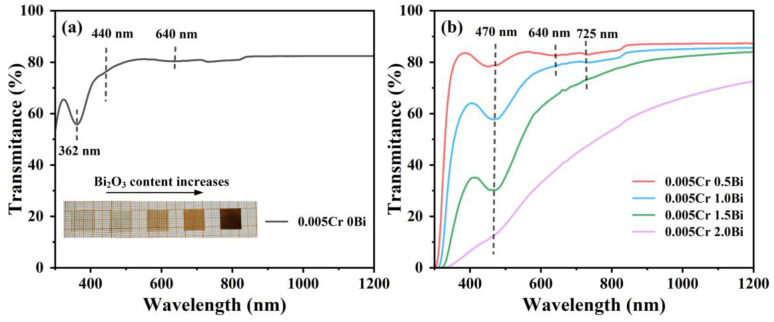
(**a**) Transmittance spectrum of 0.005Cr_2_O_3_ glass and inset are images of xBi_2_O_3_-0.005Cr_2_O_3_ glasses (x = 0, 0.5, 1, 1.5, 2). (**b**) Transmittance spectra of xBi_2_O_3_-0.005Cr_2_O_3_ glasses (x = 0.5, 1, 1.5, 2).

Figure 5a is the transmittance spectrum of the sample of 0.005 Cr_2_O_3_. In the Cr-doped sample, the absorption peak intensity of Cr ions decreases but does not disappear, as the Cr_2_O_3_ doping concentration is reduced. In Figure 5b, with the increase in the Bi_2_O_3_ content in the samples, the Bi-related absorption peak shows an enhancing trend, and there is also a weak absorption peak related to Cr ions. According to previous reports, once Bi is introduced into aluminosilicate glass at high temperature, Bi ions with different valences are generated and reach valence equilibrium in the glass structure [36]. This means that different Bi related absorption peaks may be observed in the glass samples. The weak peak at 725 nm may be attributed to the ^3^P_0_ → ^3^P_2_ transition of Bi^+^. The absorption peak at 470 nm is mainly caused by the ^4^S_3/2_ → ^2^P_1/2_ transition of Bi^0^ [37]. With increasing Bi_2_O_3_ content, the absorbance at 470 nm increases gradually, which is also a symbol of the increase in the number of Bi luminescence centers. A careful observation of the peak at 470 nm reveals that the two sides of the absorption peak are asymmetric, which may be related to the ^3^P_0_ → ^3^P_2_ transition of Bi^+^. Meanwhile, the weak absorption peak of the ^4^A_2_ → ^4^T_2_ transition of Cr^3+^ at 640 nm can also be observed, which implies that the luminescent centers of both Bi and Cr may exist simultaneously in the Bi/Cr co-doped glasses. The inset is the photo of xBi_2_O_3_-0.005Cr_2_O_3_ (x = 0, 0.5, 1, 1.5, 2) glass samples. The color of samples changes from light green to reddish brown with the addition of Bi_2_O_3_, which indicates an increase in the number of Bi luminescence centers.

**Figure 6:**
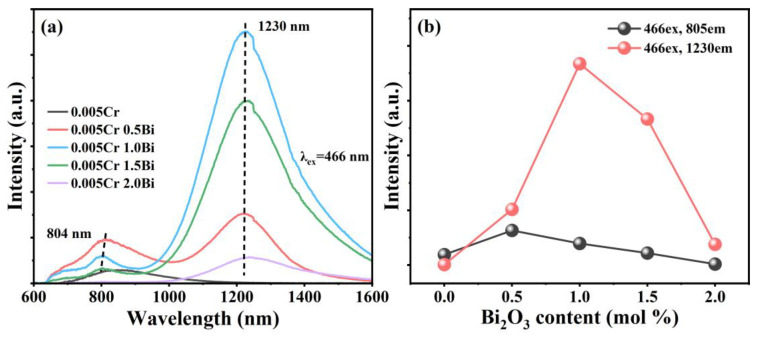
(**a**) Luminescence spectra of xBi_2_O_3_-0.005Cr_2_O_3_ glass samples excited by 466 nm (x = 0, 0.5, 1, 1.5, 2). (**b**) Dependence of luminescence intensity on the content of Bi_2_O_3_.

**Figure 7:**
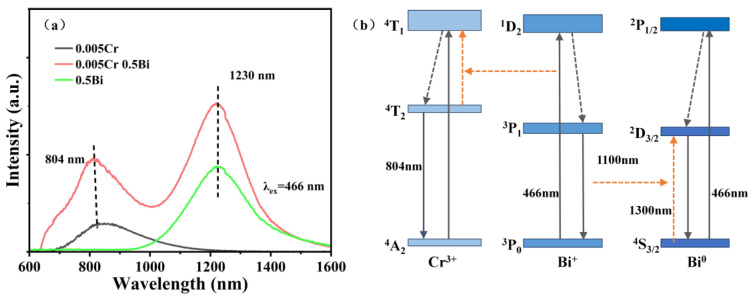
(**a**) Luminescence spectra of 0.005Cr, 0.005Cr-0.5Bi and 0.5Bi glass samples excited by 466 nm. (**b**) Simplified energy-level diagram of Cr^3+^, Bi^+^, and Bi^0^ and the possible transfer process between Cr^3+^, Bi^+^, and Bi^0^.

Figure 6a shows the emission spectra of the glass samples under 466 nm excitation. Two emission peaks in the NIR range are observed at 804 nm and 1230 nm. The peak at 1230 nm is composed of two luminescent centers at 1100 nm and 1300 nm. The emissions at 1100 nm and 1300 nm correspond to the ^3^P_1_ → ^3^P_0_ transition of Bi^+^ and the ^2^D_3/2_ → ^4^S_3/2_ transition of Bi^0^, respectively [31,38]. Under the excitation at 466 nm, Bi^+^ and Bi^0^ can be excited simultaneously, and the NIR emission leaps of Bi^+^ and Bi^0^ overlap. The emission peak at 1230 nm may be the result of the overlapping transitions of Bi^+^ and Bi^0^, which also indicates that there are two Bi-related NIR luminescence centers in the glass sample at the same time. The asymmetric luminescence peak at 1230 nm also confirms the result. By comparing Figure 2, it can be found that the emission peak around 800 nm is not observed in the sample doped with 0.5 mol Bi alone. Therefore, in the Bi/Cr co-doped samples, the emission peak near 804 nm may be the ^4^T_2_ → ^4^A_2_ transition of Cr^3+^, and the peak position blue-shifts with the change in Bi doping concentration. In other words, Bi^0^, Bi^+^, Cr^3+^ are the main luminescence centers in the glass samples, and energy exchange exists between them. The relationship between Bi doping concentration and emission peak intensity is shown in Figure 6b. With the increase in Bi doping concentration, the emission peak intensity at 1230 nm and 804 nm shows a trend of increasing first and then decreasing. The luminescence spectra of Bi-doped, Cr-doped, and Bi/Cr co-doped glass samples under 466 nm laser excitation are shown in Figure 7a. The addition of a small amount of Cr results in an increasing trend in Bi luminescence intensity. Meanwhile, compared with Bi-doped samples, the luminescence range of samples co-doped with appropriate amounts of Bi_2_O_3_ and a small amount of Cr_2_O_3_ is expanded, which involves the energy transfer between the ions mentioned above. Figure 7b is a simplified diagram of Bi^+^, Bi^0^, and Cr^3+^ derivatives. Bi^+^, Bi^0^, and Cr^3+^ are excited from the ground state to ^1^D_2_, ^2^P_1/2_, and ^4^T_1_ excitation levels by 466 nm light, and then Bi^+^ relaxes non-radiatively to the ^3^P_1_ level. During the Bi^+^ ions relaxation, Bi^+^ transfers energy to Cr^3+^. When Cr^3+^ ions transition from ^4^A_2_ level, part of the energy is consumed by non-radiative relaxation, and the rest of the energy can be emitted in the form of transition to form an emission peak at 804 nm, as shown in Figure 6a. The process of Bi^+^ transferring energy to Bi^0^ further forms the luminescence peak of 1230 nm [39]. The mechanism of energy transfer from Bi^+^ to Bi^0^ and Cr^3+^ can be described as ^3^P_1_ (Bi^+^) + ^4^S_3/2_ (Bi^0^) → ^3^P_0_ (Bi^+^) + ^2^D_3/2_ (Bi^0^) and ^1^D_2_ (Bi^+^) + ^4^T_2_ (Cr^3+^) → ^3^P_1_ (Bi+) + ^4^T_1_ (Cr^3+^), which can help the luminescence at 804 nm and 1230 nm. The luminescence peaks at these two positions can be attributed to the energy transfer induced by the co-doping of Bi and Cr, which broadens the luminescence bandwidth through this doping mechanism.

## 4. Conclusions

Bi/Cr co-doped aluminosilicate glasses were successfully prepared using a high-temperature melting process. FTIR results show that the glass structure remains stable as the Bi_2_O_3_ content increases. Emission spectra results show that the addition of low concentration Cr_2_O_3_ to Bi-doped glasses can cause the luminescence centers of Bi and Cr to exist simultaneously in the samples, which means that the luminescence range of samples is broadened by the addition of a small amount of Cr_2_O_3_. With the increase in Bi_2_O_3_ doping content, the luminescence intensity of bismuth ions can increase first. When the Bi_2_O_3_ doping concentration is higher than 1 mol%, the luminescence of Bi at 1230 nm is quenched. The luminescence peak of Cr at 804 nm has a similar trend to the luminescence peak of Bi at 1230 nm. The experimental results indicate that an appropriate increase in Bi concentration can enhance the Bi NIR emission, and there is energy exchange between Bi and Cr.

## Data Availability

The original contributions presented in the study are included in the article, further inquiries can be directed to the corresponding author.
